# Oligosaccharides as Potential Regulators of Gut Microbiota and Intestinal Health in Post-COVID-19 Management

**DOI:** 10.3390/ph16060860

**Published:** 2023-06-09

**Authors:** Kit-Leong Cheong, Shutong Chen, Bo Teng, Suresh Veeraperumal, Saiyi Zhong, Karsoon Tan

**Affiliations:** 1 Guangdong Provincial Key Laboratory of Aquatic Product Processing and Safety, Guangdong Province Engineering Laboratory for Marine Biological Products, Guangdong Provincial Engineering Technology Research Center of Seafood, Guangdong Provincial Science and Technology Innovation Center for Subtropical Fruit and Vegetable Processing, College of Food Science and Technology, Guangdong Ocean University, Zhanjiang 524088, China; klcheong@gdou.edu.cn; 2 Department of Biology, College of Science, Shantou University, Shantou 515063, China; 3 Guangxi Key Laboratory of Beibu Gulf Biodiversity Conservation, Beibu Gulf University, Qinzhou 535000, China

**Keywords:** post-COVID-19, oligosaccharide, intestinal health, gut microbiota, post-COVID-19 syndrome

## Abstract

The COVID-19 pandemic has had a profound impact worldwide, resulting in long-term health effects for many individuals. Recently, as more and more people recover from COVID-19, there is an increasing need to identify effective management strategies for post-COVID-19 syndrome, which may include diarrhea, fatigue, and chronic inflammation. Oligosaccharides derived from natural resources have been shown to have prebiotic effects, and emerging evidence suggests that they may also have immunomodulatory and anti-inflammatory effects, which could be particularly relevant in mitigating the long-term effects of COVID-19. In this review, we explore the potential of oligosaccharides as regulators of gut microbiota and intestinal health in post-COVID-19 management. We discuss the complex interactions between the gut microbiota, their functional metabolites, such as short-chain fatty acids, and the immune system, highlighting the potential of oligosaccharides to improve gut health and manage post-COVID-19 syndrome. Furthermore, we review evidence of gut microbiota with angiotensin-converting enzyme 2 expression for alleviating post-COVID-19 syndrome. Therefore, oligosaccharides offer a safe, natural, and effective approach to potentially improving gut microbiota, intestinal health, and overall health outcomes in post-COVID-19 management.

## 1. Introduction

The emergence of the severe acute respiratory syndrome coronavirus 2 (SARS-CoV-2) and the subsequent coronavirus disease 2019 (COVID-19) pandemic has had far-reaching consequences for global public health since it was first identified in Wuhan, China, in December 2019 [1]. The virus spreads primarily through respiratory droplets that are expelled when an infected individual coughs, sneezes, or talks, facilitating its rapid transmission and eventual classification as a pandemic [2]. Although the respiratory system is the main site of infection, COVID-19 can also affect other critical organs and systems in the body, including the kidneys, liver, brain, and gastrointestinal tract. The virus is a grave threat due to the broad spectrum of complications it can trigger, including acute respiratory distress syndrome, sepsis, multi-organ failure, and even death [3]. In the context of SARS-CoV-2 infection, the gastrointestinal system is also susceptible to damage, particularly in cases involving intestinal thrombosis. The virus has the ability to directly impair intestinal tissue and contribute to the formation of blood clots within the gastrointestinal tract [4]. This damage and thrombotic event can manifest as gastrointestinal symptoms and potentially lead to severe complications.

It is now widely acknowledged that a subset of people who have recovered from COVID-19 may experience persistent symptoms and complications that can affect multiple organs, leading to long-term health problems [5]. These symptoms can persist for weeks or even months, and they are collectively known as post-COVID-19 syndromes [6]. The consequences of these syndromes can be severe, as they may cause lasting harm to critical organs such as the lungs, brain, heart, and kidneys. The symptoms are diverse and can include fatigue, dyspnea, chest pain, joint pain, muscle weakness, headaches, cognitive impairment, depression, diarrhea, nausea, and abnormal pain [7]. A thorough comprehension of the long-term effects of the SARS-CoV-2 virus is crucial for accurately predicting the healthcare burden of post-COVID-19 syndrome and enhancing the quality of life for those affected. Furthermore, the development of new treatments and management strategies for post-COVID-19 syndrome is necessary to alleviate patient outcomes and mitigate the risk of long-term complications, such as organ damage and disability.

**Figure 1:**
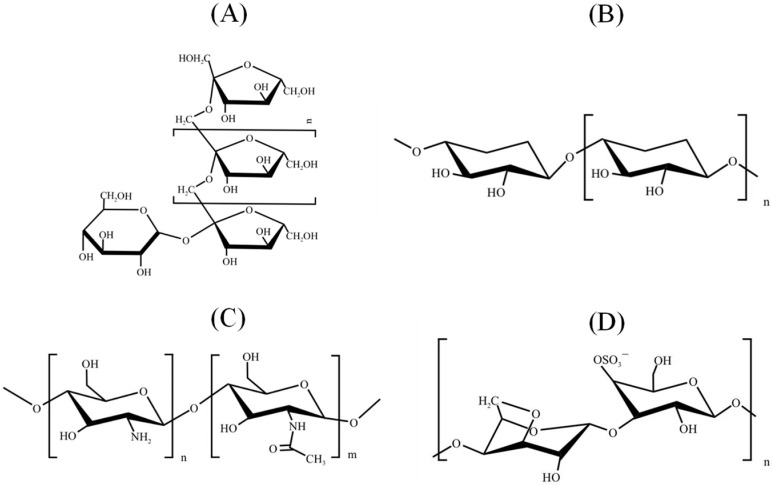
Typical chemical structures of oligosaccharides derived from natural resources, including fructo-oligosaccharide (**A**), xylo-oligosaccharides (**B**), chitosan-oligosaccharides (**C**), carrageenan-oligosaccharides (**D**), (*n* = 2–20, *n* > *m*).

Oligosaccharides are short chains of carbohydrates composed of 2–20 monosaccharide units [8]. They are present in a wide range of natural resources such as marine algae, plants, fruits, vegetables, and grains and can also be obtained through hydrolysis of polysaccharides [9,10]. Figure 1 displays the chemical structures of typical oligosaccharides obtained from natural resources, including fructo-oligosaccharide, xylo-oligosaccharides, chitosan-oligosaccharides, and carrageenan-oligosaccharides. The distinctive arrangement of monosaccharides and linkage types within each oligosaccharide type results in their unique contribution to specific biological activities. Oligosaccharides have been shown to exhibit various biological activities, including prebiotic effects, immunomodulation, and antioxidant and antiviral properties [11,12,13]. Oligosaccharides play a role as prebiotics by promoting the growth of beneficial bacteria, which can stimulate the production of mucus by goblet cells, specialized cells found in the intestinal tract [14]. This increased mucus production enhances the protective function of the intestinal mucosa by effectively capturing harmful substances, preventing their interaction with the epithelial cells [15]. Consequently, this reduces the risk of inflammation and potential damage. Oligosaccharides possess immunomodulatory and antioxidant characteristics, rendering them valuable in the treatment of diverse diseases [16,17]. In the presence of a disease, an imbalance between the generation of reactive oxygen species and the body’s antioxidant defense mechanisms often leads to oxidative stress. Oligosaccharides have the ability to alleviate oxidative stress and facilitate the recovery process [18]. They safeguard against inflammation-induced oxidative stress by diminishing the production of pro-inflammatory molecules and modulating the inflammatory response [19].

Therefore, oligosaccharides have promising potential in managing post-COVID-19 complications through their ability to modulate the intestinal flora, increase the production of functional metabolites, reduce oxidative stress, enhance immune function, improve nutrient absorption, and reinforce intestinal barrier function. The aim of this review is to provide a comprehensive summary of the existing knowledge on the potential mechanisms of action for oligosaccharides in alleviating post-COVID-19 syndromes. In addition, the review discusses the potential applications of oligosaccharides in post-COVID-19 management. The insights gained from this review could inform future research and the development of oligosaccharide interventions for managing post-COVID-19 symptoms and improving overall health outcomes.

## 2. The Negative Vicious Cycle of Post-COVID-19 Syndrome on Gastrointestinal Health

When the SARS-CoV-2 virus infects the human body, it first targets cells with angiotensin-converting enzyme 2 (ACE2) receptors on their surface, which can be found in various organs, including the lungs, heart, intestines, kidneys, pancreas, and brain [20]. Upon binding to the ACE2 receptor with its spike protein, the virus gains entry into the host cell, using its machinery to replicate and assemble new virus particles from viral proteins and genetic material [21]. Despite primarily entering through the respiratory tract, SARS-CoV-2 can also enter the body through the gastrointestinal tract due to the abundance of ACE2 receptors in the small intestine and colon lining cells [22]. This can lead to damage to the gut epithelium, resulting in inflammation, gastrointestinal symptoms, and gut dysbiosis [23]. Additionally, the virus can cause systemic inflammation and oxidative stress, resulting in changes in the gut microbiota, damage to the intestinal epithelial barrier, and intestinal permeability [24]. Although most people who contract COVID-19 recover without severe complications, there is a growing recognition of post-COVID-19 syndrome.

**Figure 2:**
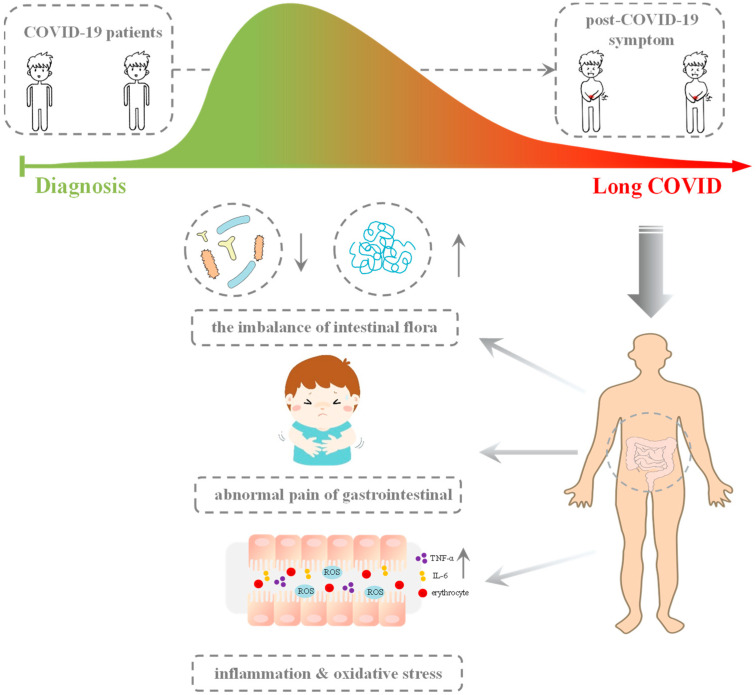
After recovering from COVID-19, patients may experience post-COVID-19 syndrome. Gut microbiota dysbiosis is a common occurrence in post-COVID-19 patients, which can result in elevated levels of inflammation and oxidative stress in the body.

The post-COVID-19 syndrome can endure for several weeks or months following the initial infection, and one of its major symptoms is gut dysbiosis, which can lead to diarrhea [25]. Studies have revealed that long COVID patients have an altered microbial profile in their gut microbiome, with decreased diversity of certain bacterial species and an increase in pathogenic bacteria growth [26]. Gut dysbiosis can have implications for immune function, mucosal function, and overall health, leading to inflammation in the gastrointestinal system, causing complications such as inflammatory bowel disease, Crohn’s disease, and ulcerative colitis [27]. Long COVID patients have elevated levels of inflammatory markers in their plasma and stool samples [28], suggesting that inflammation may play a role in gastrointestinal symptom development (as depicted in Figure 2).

Post-COVID-19 syndrome can also affect the gastrointestinal system by causing digestive symptoms such as abdominal pain, loss of appetite, and nausea [29]. The negative consequences for both gastrointestinal and systemic health can perpetuate a vicious cycle of immune dysfunction and inflammation, exacerbating the symptoms and complications of COVID-19, leading to prolonged illness courses and an increased risk of long-term complications such as chronic fatigue, cognitive impairment, and autoimmune disorders [30,31].

In short, post-COVID-19 syndrome can affect the gastrointestinal system in several ways, including gut dysbiosis, inflammation, and digestive symptoms. Therefore, maintaining intestinal health may be an important consideration in post-COVID-19 management. One potential approach is the use of prebiotics, particularly oligosaccharides (Table 1), which can selectively encourage the growth of good bacteria in the gut and help restore microbial balance, promoting a healthy immune response and supporting overall gastrointestinal health.

## 3. Oligosaccharides Attenuated Post-COVID-19 Syndrome by Modulating Gut Microbiota

The human digestive system contains a complex and diverse community of microorganisms collectively referred to as the gut microbiota. It comprises more than a thousand bacterial species, with variations in composition among individuals. Nevertheless, certain bacterial taxa, namely Bacteroidetes, Firmicutes, Actinobacteria, and Proteobacteria, are commonly found in the gut microbiota of healthy individuals [42]. The intestinal microbiota plays a crucial role in promoting human health by contributing to various physiological processes such as digestion, immune function, intestinal barrier function, and antiviral defense mechanisms [43,44]. However, dysbiosis, an imbalance of beneficial and harmful gut bacteria, has been associated with numerous health conditions, including inflammatory bowel disease, ulcerative colitis, obesity, metabolic disorders, autoimmune diseases, and mental health conditions [45]. Dysbiosis can lead to a weakened immune response and impaired gut barrier function, resulting in an increased risk of viral infections. Hence, managing dysbiosis could have significant implications for post-COVID-19 treatment [46].

The COVID-19 infection can disrupt the delicate balance of the gut microbiota, leading to dysbiosis. A previous study showed that COVID-19 patients who died had lower microbial diversity and an altered gut microbiota composition compared to the survivors. ICU patients also had a higher proportion of *Pyramidobacter* and *Eremococcus* and a lower proportion of *Collinsella* and *Eubacterium ventriosum* groups [47]. The imbalances in gut microbiota can persist even after recovering from the virus, leading to potential long-term health consequences. At 6 months’ follow-up, microbial diversity and richness in patients with post-COVID-19 were significantly lower than in healthy individuals [48]. Several recent studies have demonstrated the positive impact of oligosaccharides on gut microbiota diversity and balance [15]. Our previous research has shown that *Gracilaria lemaneiformis* oligosaccharides can alleviate the severity of dextran sulphate sodium-induced colitis by preventing mice from inflammatory infiltration and maintaining gut balance [49]. In addition, in vitro studies using human feces fermentation have found that *G. lemaneiformis* and *Saccharina japonica* oligosaccharides can modulate the composition and diversity of gut microorganisms [50,51]. Furthermore, existing clinical research has shown that intervention with fructo-oligosaccharides has the potential to modulate the communication between the microbiota and the brain through the gut–brain axis. This intervention may lead to an improvement in gut diversity and a reduction in hyper-serotonergic state and dopamine metabolism disorder, both of which are frequently observed in individuals with autism spectrum disorder [52]. 

According to a previous study, there exists an inverse correlation between *Bacteroides dorei*, *Bacteroides thetaiotaomicron*, *Bacteroides massiliensis*, and *Bacteroides ovatus* and SARS-CoV-2 load in fecal samples obtained from patients [53]. *Bacteroides* is a genus of bacteria that is commonly present in the human gut flora and plays an important role in breaking down and metabolizing complex carbohydrates. It is noteworthy that *Bacteroides* have a significant portion of their genome dedicated to oligosaccharides utilization. This is due to the presence of genes that encode for enzymes, also known as carbohydrate-active enzymes (CAZymes), which can break down oligosaccharides, as well as proteins involved in transport and regulation that are organized into polysaccharide utilization loci [54,55]. Several studies suggest that *Bacteroides* is involved in immune function and inflammation regulation [56]. For instance, *Bacteroides* has been shown to improve ulcerative colitis [57] and alleviate inflammation induced by obesity [58]. *Bacteroides* spp. are capable of utilizing oligosaccharides as nutrients due to their complex enzymatic machinery, making them thrive in oligosaccharide-enriched environments [59]. Consequently, a high intake of dietary fiber or oligosaccharides may help in weight maintenance and reduce the risk of inflammatory diseases [60]. However, in the absence of fiber in the diet, *Bacteroides* spp., particularly *Bacteroides thetaiotaomicron*, may selectively adapt their transcriptional responses, leading to the degradation of host mucus glycans as a nutrient, which can cause thinning of the mucus layer [61]. Nevertheless, the intake of dietary oligosaccharides can protect the mucus layer in the gut by enhancing the growth of *Bacteroidetes*, which preferentially use oligosaccharides as a source of energy, rather than digesting the mucus layer as a nutrient [62]. In a randomized, double-blind, placebo-controlled study, it was found that administering a mixture of galacto-oligosaccharides to elderly volunteers (aged 65–80 years) resulted in significant increases in *Bacteroides-Prevotella*. Furthermore, this administration was also shown to increase the levels of IL-10, IL-8, natural killer cell activity, and C-reactive protein, while reducing the level of IL-1β [63]. Recent research has revealed that 76% of patients with post-acute COVID-19 syndrome experience common symptoms, such as fatigue, poor memory, and hair loss, six months after the onset of the disease. Analysis of the gut microbiome in these patients has also shown higher levels of *Bacteroides vulgatus*, suggesting a potential link between this bacterial strain and the development of post-acute COVID-19 syndrome symptoms [48]. Studies have demonstrated that *B. vulgatus* exhibits potent immune-modulating properties, resulting in the prevention of colitis induction in various mouse models of experimental colitis [57]. Additionally, pectic oligosaccharides or inulin can differentially modulate the progression of leukemia and associated metabolic disorders by specifically increasing the abundance of *B. vulgatus* [64].

The Firmicutes phylum is one of the largest bacterial phyla, consisting of over 200 genera, including *Staphylococcus*, *Lactobacillus*, *Bacillus*, *Ruminococcus*, and *Clostridium* [65]. However, an excessive abundance of Firmicutes has been linked to increased inflammation and is positively associated with dysbiosis, while lower numbers of Firmicutes are considered more desirable [66]. In post-COVID-19 patients, an imbalance in the gut microbiota has been identified by an elevation in the prevalence of Firmicutes. Previous studies have proved that COVID-19 disease severity is linked to a higher abundance of the phylum Firmicutes, such as the *Coprobacillus* genus, *Clostridium ramosum*, and *Clostridium hathewayi* species [53]. Moreover, studies in humans have shown an inverse relationship between the severity of the disease and the abundance of *Faecalibacterium* and *Roseburia* [28,67]. *Faecalibacterium prausnitzii*, a species within the Firmicutes phylum and member of the Ruminococcaceae family, is among the most commonly detected species in the human gut flora and is a primary source of butyrate in the colon. Studies have shown that *F. prausnitzii* can grow by acquiring and degrading various β-mannooligosaccharides, and the growth of this species can be increased by β-mannooligosaccharides [68]. Cross-feeding is a fascinating type of metabolic interaction that takes place among commensal microbes in the gut, in which certain organisms with the ability to process poly-/oligo-saccharides can sustain other members of their community. This phenomenon may arise from competition for available carbohydrates, differential utilization of released composition of oligosaccharides, or additional processing of fermentation byproducts [69]. It has been observed that cross-feeding with *Bacteroides ovatus* and *Roseburia intestinalis* is required for the degradation of β-mannan into β-mannooligosaccharides, which can be further utilized by *F. prausnitzii* [68]. These cross-feeding interactions have a beneficial impact on intestinal health. Some of the species of *Bacteroides* and *Firmicutes* can break down oligosaccharides into simple carbohydrates, which can be shared and used by other bacteria through cross-feeding. Cross-feeding has also been observed in the fermentation of substrates such as xylan and xylo-oligosaccharides by co-cultures of *Bacteroides* and *R. intestinalis*. The transport protein of *R. intestinalis* shows a preference for xylo-oligomers consisting of 4–5 units, while *Bacteroides* primarily degrades xylan into xylo-oligosaccharides [70]. After *B. ovatus* DSMZ 1896 was grown in galactomannan-supplemented media, β-manno-oligosaccharides with a degree of polymerization (DP) of 2–4, which are breakdown products of polysaccharides, were left over. These breakdown products were found to promote the proliferation of *Lactiplantibacillus plantarum* WCFS1 (primarily DP2 and DP3) and *Bifidobacterium adolescentis* DSMZ 20,083 (mostly DP3), while lactate and acetate were produced as a result of this process [71].

Therefore, maintaining a healthy gut microbiota is essential in the post-COVID-19 management. It can help boost the immune response, reduce the severity of the illness, and shorten the duration of the virus infection. Oligosaccharide is a potential strategy to promote a healthy gut microbiota used to promote the growth of advantageous gut microbiota, supporting overall health and well-being in the post-COVID-19 management.

## 4. The Role of Oligosaccharide in the Gut Microbiota-Derived Short-Chain Fatty Acids and Facilitate Post COVID-19 Syndromes

The intestinal microbiota plays an important role in maintaining gut and overall health by producing gut-derived metabolites through the fermentation of non-digestible carbohydrates, such as oligosaccharides. These metabolites, primarily produced in the colon, include short-chain fatty acids (SCFAs), such as acetate, propionate, and butyrate [15,72,73]. SCFAs serve as an essential energy source for colonic epithelial cells and promote the integrity of the intestinal barrier, regulate tight junction proteins, prevent oxidative stress, and modulate immune function. SCFAs exert their effects by attaching to particular receptors on intestinal epithelial cells’ surface, which includes G-protein-coupled receptors 41 and 43 (GPR41 and GPR43), also known as free fatty acid receptors 2 and 3 (FFAR-2 and FFAR-3), respectively [74]. The activation of GPR41 and GPR43 by SCFAs has been shown to have several physiological effects, such as regulating gut motility, reducing inflammation, and promoting colonic epithelial cell proliferation and differentiation. Moreover, SCFAs and GPR41 signaling are linked to regulating insulin sensitivity and energy homeostasis. In addition to GPR41 and GPR43, SCFAs also stimulate receptors such as GPR109a and OR51E2 [75]. The expression of GPR109a is observed in the host’s intestinal epithelial cells and various immune cells, including macrophages, dendritic cells, monocytes, and neutrophils, while OR51E2 is expressed in the colon and rectum [76]. Activation of GPR109a and OR51E2 by SCFAs leads to a range of downstream effects, including the release of inflammatory cytokines, regulation of immune cell proliferation and differentiation, and modulation of gut hormone secretion [77].

The analysis of fecal metabolites showed that COVID-19 patients had significantly lower concentrations of SCFAs both before and after disease resolution. Moreover, the impairment of SCFA production in the intestinal microbiome continued for more than 30 days after recovery [78], which led to a reduction in SCFA-producing bacteria for COVID-19 and post-COVID-19 patients [79,80]. The most well-known SCFA-producing bacteria belong to the Firmicutes and Bacteroidetes phyla, including members of the genera *Bacteroides*, *Bifidobacterium*, *Clostridium*, *Lactobacillus*, *Prevotella*, and *Ruminococcus*. Each of these bacteria has a unique metabolic profile that influences the type and amount of SCFAs they produce. Evidence suggests that oligosaccharides can increase the production of SCFA-producing bacteria and SCFAs. For example, cranberry arabino-xyloglucan and pectic oligosaccharides promoted the growth of *Lactobacillus* species, such as *Lactobacillus acidophilus*, *Lactobacillus plantarum*, and *Lactobacillus fermentum*, and also increased the production of SCFAs, with butyrate being the most prominent SCFA [81]. Galacto-oligosaccharides from *Lupinus albus* were found to increase SCFA production, particularly butyrate, by increasing the relative abundance of Firmicutes phylum by 110% compared to the untreated group with ulcerative colitis [82]. The main butyrate-producing bacteria include *Faecalibacterium prausnitzii*, *Clostridium butyricum*, *Eubacterium rectale*, *Roseburia* spp., *Eubacterium hallii*, *Akkermansia muciniphila*, *Bifidobacterium* spp., and *Lactobacillus* spp.

SCFAs play a critical role in regulating the immune response and reducing inflammation. They interact with various immune cells such as dendritic cells, macrophages, and T cells [83], thereby influencing the production of pro-inflammatory cytokines such as interleukin (IL)-1β, IL-6, and tumor necrosis factor (TNF)-α. SCFAs also promote the production of anti-inflammatory cytokines like IL-10, while promoting the differentiation and function of regulatory T cells, which are critical in preserving immune homeostasis and preventing autoimmune diseases [84]. Additionally, SCFAs can modulate the expression of immune receptors and signaling pathways, including Toll-like receptors (TLRs) and nuclear factor kappa B (NF-κB), which are involved in regulating the immune response and inflammation.

Acetate is a critical component in energy metabolism, serving as a substrate for gluconeogenesis in the liver and a source of energy for other tissues. It has also been suggested that acetate may reduce the generation of pro-inflammatory cytokines and enhance cytokine production in T cells, potentially improving the immune response during glucose restriction caused by infections or other stressful situations [85]. These results have important implications for the role of acetate in immune function, particularly in the context of COVID-19 infections. Post-COVID-19 patients show evidence of T cell dysfunction and impaired immune responses, contributing to long-term symptoms and poor outcomes [86]. Studies have demonstrated that oligosaccharides act as prebiotics, promoting the growth of acetate-producing bacteria and subsequently increasing acetate production. For instance, the addition of fructo-oligosaccharides has been shown to elevate the relative abundance of bacterial genera such as *Bacteroides*, *Anaerostipes*, and *Lactobacillus*, which are known to facilitate acetate production [87]. 

Butyrate has been shown to enhance the immune response against influenza infection in mice by increasing the production of Ly6c-patrolling monocytes in the bone marrow, which can clear the virus by migrating to the lungs. In addition, a high-fiber diet that raises butyrate levels can enhance antiviral function by promoting the shift of CD8+ T cells in the lungs towards increased fatty acid oxidation and decreased glycolysis, leading to improved antiviral activity [88]. Interestingly, xanthan gum oligosaccharides and gellan gum oligosaccharides have been found to increase the production of butyrate-producing bacteria, such as *Lachnospiraceae*, and butyric acid production in an in vitro fermentation assay using human fecal inocula [89]. Butyrate deficiency has been associated with altered bacterial networks and increased symptoms of fatigue in patients with myalgic encephalomyelitis/chronic fatigue syndrome [90]. Recent studies have also suggested that short-chain fatty acids (SCFAs), including butyrate, may have a beneficial effect on post-COVID-19 symptoms such as fatigue and cognitive impairment [91]. Additionally, oligosaccharides derived from *Codonopsis pilosula* have been shown to have antifatigue and antihypoxia activities in mice [92]. Neoagaro-oligosaccharides administration has been shown to alleviate depression induced by chronic restraint stress in mice. This is achieved by increasing levels of brain-derived neurotrophic factor and 5-hydroxytryptamine in the brain, reversing the decrease in short-chain fatty acid levels in the cecum of depressed mice and mitigating gut microbiota dysbiosis [93]. Therefore, oligosaccharides may have a beneficial effect on fatigue and cognitive impairment in general.

To potentially improve post-COVID-19 syndrome through dietary interventions, promoting the production of SCFAs via oligosaccharides has been suggested, as previously mentioned. Similarly to a previous report, it has been recommended to consume viscous and fermentable fibers such as β-glucan and arabinoxylans from whole grains and pectin from fruits, vegetables, and legumes, as they have a prebiotic effect on SCFA-producing bacteria [94,95]. The Mediterranean diet is an example of a fiber-rich diet that impacts on the structure and function of the intestinal microbiota and the production of SCFAs [96]. Postbiotic interventions, such as bioactive compounds produced by live microorganisms during fermentation that confer health benefits on the host, particularly SCFAs, could also be used to treat post-COVID-19 syndrome [97]. As indicated in Table 2, the SCFAs have the potential to be beneficial for post-COVID-19 syndrome.

In short, the use of oligosaccharides interventions or supplementation to produce gut-derived metabolites, particularly short-chain fatty acids (SCFAs), has been shown to modulate immune response and reduce inflammation. There is evidence to suggest their potential use in alleviating post-COVID-19 symptoms, and they have emerged as a potential therapeutic target for alleviating post-COVID-19 symptoms.

## 5. Role of Oligosaccharides and Gut Microbiota-Derived Bile Salts in Post COVID-19 Recovery

The liver synthesizes primary bile acids that are conjugated to taurine and glycine before being secreted into the gut. However, the intestinal microbiota also plays a significant role in bile salt metabolism [106]. Unabsorbed bile salts in the small intestine enter the large intestine where gut bacteria metabolize them. This conversion of primary bile acids, including cholic and chenodeoxycholic acid, into secondary bile acids, such as deoxycholic and lithocholic acid, is responsible for up to half of bile acid metabolism in the human body [107]. This microbial conversion has numerous implications, including evidence suggesting that microbial metabolites such as bile acids play a crucial role in the replication of enteric viruses, such as porcine sapoviruses, porcine enteric calicivirus, and noroviruses [108]. Moreover, secondary bile acids have different physical properties from primary bile acids, which can impact host metabolism and inflammation [109].

Research has shown that COVID-19 infection patients have lower contents of secondary bile acids, such as deoxycholic acid and ursodeoxycholic/hyodeoxycholic acid, compared to COVID-19-negative patients. These secondary bile acids are produced through gut microbiome metabolism, and their decreased levels suggest dysregulation of bile acid metabolism in COVID-19 patients [109]. This dysregulation could contribute to post-COVID-19 symptoms, including fatigue. Studies have demonstrated that oligosaccharides can modulate bile acid composition to enhance energy metabolism and lipid metabolism. For example, when hyperlipidemic mice were supplemented with mannan oligosaccharides in their diet, an increase in secondary bile acids was observed, resulting in reduced atherosclerosis development [110]. Similarly, administering ι-carrageenan tetrasaccharide to insulin-resistant mice on a high-fat, high-sucrose diet led to an increase in bile acid levels in serum, liver, and feces [111]. 

Bile acids have been shown to exhibit anti-inflammatory characteristics in various settings [112] and may serve as a means to regulate the inflammatory response in individuals with post-COVID-19 symptoms. Ursodeoxycholic acid, for example, has been found to regulate the innate immune response by activating signaling pathways such as NF-κB [113]. Additionally, ursodeoxycholic acid reduces the activation and proliferation of T cells, which can help suppress the immune response in specific circumstances [114]. Ursodeoxycholic acid also exhibits anti-inflammatory effects and can inhibit pro-inflammatory cytokines, including IL-6 and IL-1β [115]. Furthermore, Wahlström et al. have noted that bile acid modulation can impact the microbiota both directly and indirectly and activate innate immune genes in both the gut and lung, indicating a potential crosstalk between them [116]. The farnesoid X receptor (FXR) has emerged as an important nuclear receptor that regulates bile acid homeostasis and is activated by various bile acids [117]. FXR is expressed in different tissues of the body, including the lung, liver, and intestine. Targeting the FXR pathway has been suggested as a potential therapeutic approach for COVID-19 [118]. FXR activation has been found to have anti-inflammatory effects, possibly by regulating cytokine production and inhibiting NF-κB signaling [119]. Moreover, research has investigated FXR agonists, such as obeticholic acid, for their therapeutic implications in COVID-19. Obeticholic acid has been shown to have anti-inflammatory effects and improve respiratory function in animal models of lung injury, indicating its potential to alleviate the cytokine storm and lung injury associated with severe COVID-19 [120]. As such, targeting bile acid metabolism may have important implications for managing post-COVID-19 symptoms such as fatigue and persistent chronic inflammation. 

## 6. The Role of Oligosaccharides in Modulating Gut Microbiota and ACE2 Expression for Alleviating Post-COVID-19 Syndrome

**Figure 3:**
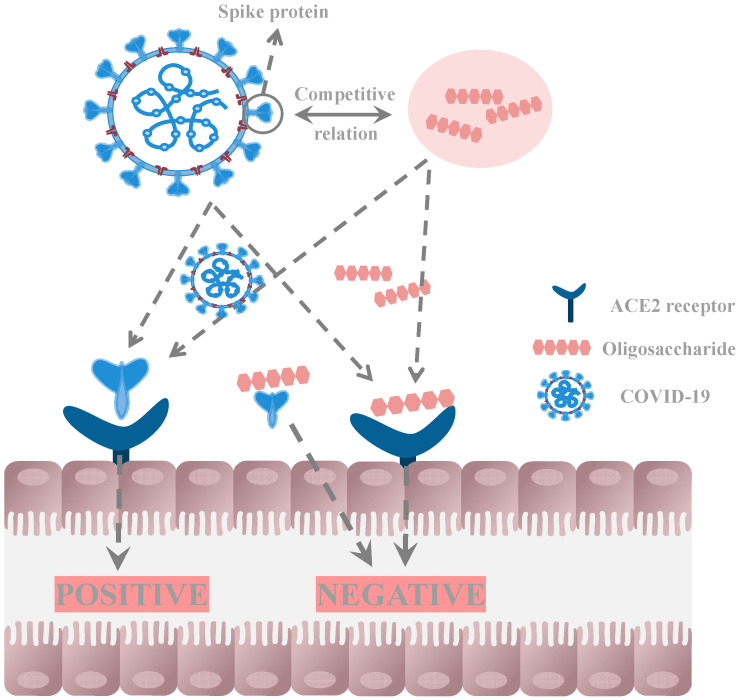
Oligosaccharides have the ability to interact with both the spike protein and intestinal ACE2, leading to inhibition of COVID-19 virus replication.

SARS-CoV-2 gains entry into host cells by attaching to the ACE2 receptor on the cell surface. ACE2 is present on various cells in the human body, including those of the respiratory and gastrointestinal tracts [121]. Once inside the cell, the virus uses the host cell’s machinery to replicate and spread. Polysaccharides and oligosaccharides derived from bacterial, fungal, and marine algal sources are natural compounds that exhibit bioactive properties capable of enhancing the immune system, inhibiting viral replication and infectivity, and providing protection against viral infections [122]. Current research has prioritized the exploration of sulfated polysaccharides and oligosaccharides as promising approaches to combat SARS-CoV-2. For instance, heparin, an anticoagulant medication, has demonstrated remarkable effectiveness at the nanomolar level in preventing the transmission of SARS-CoV-2 [123]. This is accomplished by inhibiting viral attachment and reducing the formation of blood clots. Furthermore, sulfated polysaccharides derived from plants and marine organisms have displayed encouraging inhibitory effects against the virus in laboratory experiments, effectively diminishing viral replication and reducing infectivity [124]. These molecules can bind to both ACE2 and the spike protein of SARS-CoV-2, which facilitates the virus’s attachment to ACE2 [125,126]. By doing so, sulfated oligosaccharides can competitively prevent the virus from entering host cells, potentially reducing the severity of post-COVID-19 symptoms (Figure 3).

Post-COVID-19 patients may harbor a small viral load in their bodies even after recovering from the acute phase, which can activate intestinal ACE2 receptors and cause gastrointestinal symptoms such as diarrhea [127]. Previous studies using gnotobiotic rats lacking natural gut microbiota found that the presence of gut microbiota is associated with increased expression of ACE2 mRNA in the colon. This increased expression of ACE2 could increase susceptibility to SARS-CoV-2 infection and affect disease severity [128]. The gut microbiota regulates ACE2 expression, with a healthy microbiome promoting higher ACE2 expression and less severe disease. Certain bacteria, such as *Bacteroides dorei*, *Bacteroides ovatus*, *Bacteroides thetaiotaomicron*, and *Bacteroides massiliensis*, downregulate ACE2 expression in murine models, highlighting the interrelationship between the gut microbiome, ACE2 expression, and viral infection [53]. The effect of *Firmicutes* species on ACE-2 receptor expression is not consistent. However, recent studies have indicated that modulating the gut microbiota composition by increasing the abundance of Bacteroidetes and decreasing the levels of Firmicutes may have a beneficial impact in inhibiting the entry of SARS-CoV-2 through the downregulation of ACE2 expression in the intestinal epithelial cells [129].

The gut–lung axis is a two-way communication pathway that facilitates the exchange of information between the gut microbiota and the respiratory system [130]. Growing evidence shows that the gut microbiota influences respiratory health, while the respiratory system affects gut microbiota composition. The immune system, critical in both the gut and lungs, is believed to mediate the gut–lung axis, along with ACE2 expression in both organs [131]. Studies reveal that the gut microbiota modulates ACE2 expression in the respiratory system, and that treatment with *Lactobacillus rhamnosus* can increase ACE2 expression in the lungs, mitigate influenza virus-induced lung injury, and improve lung function [132]. Various oligosaccharides, including fructo-oligosaccharides, xylooligosaccharides, galacto-oligosaccharides, and pectin-oligosaccharides, have been shown to selectively promote the growth of beneficial gut bacteria such as *Bifidobacteria* and *Lactobacilli*. These probiotic-derived bacteria produce molecules such as lipopeptides, including subtilisin from *Bacillus amyloliquefaciens*, curvacin A from *Lactobacillus curvatus*, sakacin P from *Lactobacillus sakei*, and lactococcin Gb from *Lactococcus lactis*, which possess a higher binding affinity to human ACE2 [133]. By competitively inhibiting the action of these probiotic-derived molecules, SARS-CoV-2’s mandatory connection with host epithelial cells expressing ACE2 for entry and reproduction is prevented. Moreover, research has demonstrated that the gut microbiota produces SCFAs that can influence the expression of intestinal ACE2. Brown et al. demonstrated that colonization with Clostridia-enriched bacteria resulted in a significant increase in fecal propionate and butyrate, as well as a decrease in ACE2 expression in the intestines and lungs of specific pathogen-free mice [134]. Similarly, other research has shown that butyrate treatment can decrease the expression of ACE2, along with various other genes related to host defense and immune response [135]. These findings suggest that SCFAs may play a role in inhibiting SARS-CoV-2 entry into host cells by lowering ACE2 expression. Therefore, targeting and modulating the gut microbiome using oligosaccharides could be a potential strategy to reduce post-COVID-19 symptoms.

## 7. Conclusions

The global outbreak of COVID-19 has had a profound influence on the lifestyles of individuals across the globe and can cause long-term health effects, including post-COVID-19 syndrome. This syndrome may persist for weeks or months and can include symptoms such as gut dysbiosis, diarrhea, fatigue, and abnormal pain. Disruptions in the gut microbiota can contribute to various gastrointestinal symptoms and an increased risk of infections. Furthermore, dysbiosis in the gut microbiota can lead to increased inflammation and oxidative stress, which can contribute to chronic symptoms and the development of post-COVID-19 syndrome. Therefore, balancing the intestinal microbiota through dietary interventions, probiotics, prebiotics, and fecal microbiota transplantation has emerged as a promising therapeutic approach for alleviating post-COVID-19 symptoms.

Personalized nutrition is an emerging field that could provide new opportunities for promoting gut health in post-COVID-19 management. Combining oligosaccharides with other probiotics and prebiotics could lead to more significant improvements in gut health and immune function. Oligosaccharides are derived from natural resources and are non-toxic, making them a convenient and low-cost method for promoting gut health and reducing the risk of gastrointestinal symptoms and infections. They can stimulate the growth of beneficial gut bacteria while inhibiting the growth of harmful bacteria, promoting the production of functional metabolites such as short-chain fatty acids (SCFAs). SCFAs have numerous health benefits, including immune-modulation and anti-inflammation. Bile salts, another important gut-derived metabolite, may also have antioxidant and anti-inflammatory capabilities that can help reduce inflammation and oxidative stress. Moreover, the gut microbiota and ACE2 receptors play crucial roles in the pathogenesis of COVID-19 and may also be implicated in the development of post-COVID-19 syndrome. Oligosaccharides may modulate the gut flora and the expression of ACE2 in the gut, highlighting the potential importance of gut microbiota modulation in post-COVID-19 symptom management.

However, further research is necessary to determine the optimal dosage and timing of oligosaccharide supplementation in post-COVID-19 management. Additionally, it is essential to consider the source and type of oligosaccharide, as different types can have varying effects on the gut microbiota. Future research should focus on identifying the specific oligosaccharides that are most effective in promoting gut health. In conclusion, oligosaccharides are potential regulators of gut microbiota and intestinal health in post-COVID-19 management.

## Figures and Tables

**Table 1:** Potential effect of oligosaccharides in reducing post-COVID-19 syndromes and their related mechanisms.

Oligosaccharides	Methods	Results and Mechanisms	Ref.
Arabinoxylan–oligosaccharides	20 healthy volunteers consumed arabinoxylan–oligosaccharides (15 g/d in the first week, 30 g/d in the second week)	There has been an increase in colonic fermentation, resulting in a higher concentration of stool moisture.	[32]
Dragon fruit oligosaccharides	107 healthy adults, divided into 3 groups that received oligosaccharides in drinking waterdoses of 4 and 8 g/day, compared to the placebo group for 4 weeks	Enhancing IgA levels, promoting a healthy gut microbiota, and reducing harmful bacteria.	[33]
Fructo-oligosaccharides	56 people aged 18–75 years with spinal cord injury during inpatient rehabilitation, who require antibiotics, will be given probiotics or placebo randomly	Decreased occurrences of nausea and diarrhea, leading to an improved quality of life.	[34]
Fructo-oligosaccharides	50 patients with type-2 diabetes were randomly assigned to the symbiotic or placebo groups to receive one sachet daily for 12 weeks	There were no changes observed in the levels of serum triglycerides, total cholesterol, HDL-cholesterol, and LDL-cholesterol. However, there was an improvement in metabolic factors and a reduction in inflammation.	[35]
Fructo-oligosaccharides	Placebo-controlled, randomized, double blind design in 60 older participants aged 65 and over	There was improvement observed in two frailty criteria, specifically exhaustion and handgrip strength.	[36]
Fructo-oligosaccharides	27 middle-aged subjects were randomized to take synbiotic (Bifidobacterium animalis lactis and oligosaccharides) or placebo for 30 days	There was a reduction in inflammatory markers along with an improvement in gut disorders.	[37]
Galacto- oligosaccharides	Double-blind, placebo-controlled, crossover study, subjects from the general population	The intervention resulted in improvements in bloating, flatulence, and abdominal pain. It exhibited high selectivity towards bifidobacteria, while also modulating the metabolic and compositional aspects of the microbiota.	[38,39,40]
Xylo-oligosaccharides	Prebiotic (xylo-oligosaccharide, 8 g/d), probiotic (Bifidobacterium animalis) or synbiotic was given to healthy adults (25–65 years) for 21 days	The intervention promoted bifidogenesis, resulting in an increase in bifidobacteria population. It also improved aspects of the plasma lipid profile, and effectively modulated markers of immune function in healthy adults.	[41]

**Table 2:** Typical gut microbiota metabolites and their roles in health.

Metabolites	Mechanism	Specific Functions	Ref.
Bile acid	Resistance to *Clostridium difficile* infection	Provided protection against antibiotic-induced diarrhea.	[98]
Bile acid	Balancing the gut microbiota and metabolism of bile acid	Anti-inflammatory effects	[99]
Bile acid, ursodeoxycholic acid	Attenuated release of proinflammatory cytokines from colonic epithelial cells	Anti-inflammatory effects	[100]
Niacin	Acting as a GPR109A agonist in immune cells	Anti-inflammatory effects	[101]
SCFAs, butyrate	Increase the expression of tight junction proteins	Maintains homeostasis of the central nervous system.	[102]
SCFAs	SCFA translocate from the intestinal mucosa to the systemic circulation	Modulates the gut microbiota and regulates the maturation and function of microglia in the central nervous system.	[103]
SCFAs, acetate	Enhanced T-regulatory cell numbers and function. Acetate increases acetylation at the Foxp3 promoter and inhibits HDAC9.	Offered protection against the development of airway disease in the offspring.	[104]
SCFAs, propionate	Signaling through the receptor GPR41	Provided protection against allergic inflammation in the lungs.	[105]

## Data Availability

Not applicable.

## References

[1] Yang X., Yu Y., Xu J., Shu H., Xia J.A., Liu H., Wu Y., Zhang L., Yu Z., Fang M. (2020). Clinical course and outcomes of critically ill patients with SARS-CoV-2 pneumonia in Wuhan, China: A single-centered, retrospective, observational study. Lancet Resp. Med..

[2] Tang J.W., Bahnfleth W.P., Bluyssen P.M., Buonanno G., Jimenez J.L., Kurnitski J., Li Y., Miller S., Sekhar C., Morawska L. (2021). Dismantling myths on the airborne transmission of severe acute respiratory syndrome coronavirus-2 (SARS-CoV-2). J. Hosp. Infect..

[3] Liu J., Li Y., Liu Q., Yao Q., Wang X., Zhang H., Chen R., Ren L., Min J., Deng F. (2021). SARS-CoV-2 cell tropism and multiorgan infection. Cell Discov..

[4] Wu X., Jing H., Wang C., Wang Y., Zuo N., Jiang T., Novakovic V.A., Shi J. (2022). Intestinal damage in COVID-19: SARS-CoV-2 infection and intestinal thrombosis. Front. Microbiol..

[5] Nalbandian A., Sehgal K., Gupta A., Madhavan M.V., McGroder C., Stevens J.S., Cook J.R., Nordvig A.S., Shalev D., Sehrawat T.S. (2021). Post-acute COVID-19 syndrome. Nat. Med..

[6] Crook H., Raza S., Nowell J., Young M., Edison P. (2021). Long covid—Mechanisms, risk factors, and management. BMJ.

[7] Raveendran A.V., Jayadevan R., Sashidharan S. (2021). Long COVID: An overview. Diabetes Metab. Synd..

[8] Naveed M., Phil L., Sohail M., Hasnat M., Baig M.M.F.A., Ihsan A.U., Shumzaid M., Kakar M.U., Mehmood Khan T., Akabar M.D. (2019). Chitosan oligosaccharide (COS): An overview. Int. J. Biol. Macromol..

[9] de Moura F.A., Macagnan F.T., da Silva L.P. (2015). Oligosaccharide production by hydrolysis of polysaccharides: A review. Int. J. Food Sci. Technol..

[10] Xie X.-T., Cheong K.-L. (2022). Recent advances in marine algae oligosaccharides: Structure, analysis, and potential prebiotic activities. Crit. Rev. Food Sci. Nutr..

[11] Wang M., Veeraperumal S., Zhong S., Cheong K.-L. (2023). Fucoidan-derived functional oligosaccharides: Recent developments, preparation, and potential applications. Foods.

[12] Moreno F.J., Corzo N., Montilla A., Villamiel M., Olano A. (2017). Current state and latest advances in the concept, production and functionality of prebiotic oligosaccharides. Curr. Opin. Food Sci..

[13] Guo Z., Wei Y., Zhang Y., Xu Y., Zheng L., Zhu B., Yao Z. (2022). Carrageenan oligosaccharides: A comprehensive review of preparation, isolation, purification, structure, biological activities and applications. Algal Res..

[14] Yu B., Wang M., Teng B., Veeraperumal S., Cheung P.C.-K., Zhong S., Cheong K.-L. (2023). Partially acid-hydrolyzed porphyran improved dextran sulfate sodium-induced acute colitis by modulation of gut microbiota and enhancing the mucosal barrier. J. Agric. Food. Chem..

[15] Zhang N., Jin M., Wang K., Zhang Z., Shah N.P., Wei H. (2022). Functional oligosaccharide fermentation in the gut: Improving intestinal health and its determinant factors-A review. Carbohydr. Polym..

[16] Cheong K.-L., Li J.-K., Zhong S. (2022). Preparation and structure characterization of high-value *Laminaria digitata* oligosaccharides. Front. Nutr..

[17] Wang T., Tao Y., Lai C., Huang C., Ling Z., Yong Q. (2022). Influence of glycosyl composition on the immunological activity of pectin and pectin-derived oligosaccharide. Int. J. Biol. Macromol..

[18] Bi D., Yang X., Lu J., Xu X. (2022). Preparation and potential applications of alginate oligosaccharides. Crit. Rev. Food Sci. Nutr..

[19] Zheng L.-X., Liu Y., Tang S., Zhang W., Cheong K.-L. (2023). Preparation methods, biological activities, and potential applications of marine algae oligosaccharides: A review. Food Sci. Hum. Well..

[20] Oudit G.Y., Wang K., Viveiros A., Kellner M.J., Penninger J.M. (2023). Angiotensin converting enzyme 2-at the heart of the COVID-19 pandemic. Cell.

[21] Behl T., Kaur I., Bungau S., Kumar A., Uddin M.S., Kumar C., Pal G., Sahil, Shrivastava K., Zengin G. (2020). The dual impact of ACE2 in COVID-19 and ironical actions in geriatrics and pediatrics with possible therapeutic solutions. Life Sci..

[22] Bourgonje A.R., Abdulle A.E., Timens W., Hillebrands J.-L., Navis G.J., Gordijn S.J., Bolling M.C., Dijkstra G., Voors A.A., Osterhaus A.D. (2020). Angiotensin-converting enzyme 2 (ACE2), SARS-CoV-2 and the pathophysiology of coronavirus disease 2019 (COVID-19). J. Pathol..

[23] Zhang H., Li H.-B., Lyu J.-R., Lei X.-M., Li W., Wu G., Lyu J., Dai Z.-M. (2020). Specific ACE2 expression in small intestinal enterocytes may cause gastrointestinal symptoms and injury after 2019-nCoV infection. Int. J. Infect. Dis..

[24] Fodor A., Tiperciuc B., Login C., Orasan O.H., Lazar A.L., Buchman C., Hanghicel P., Sitar-Taut A., Suharoschi R., Vulturar R. (2021). Endothelial dysfunction, inflammation, and oxidative stress in COVID-19—Mechanisms and therapeutic targets. Oxid. Med. Cell Longev..

[25] Freire M.P., Oliveira M.S., Magri M.M.C., Tavares B.M., Marinho I., Nastri A.C.D.S.S., Filho G.B., Levin A.S. (2022). Frequency and factors associated with hospital readmission after COVID-19 hospitalization: The importance of post-COVID diarrhea. Clinics.

[26] Alharbi K.S., Singh Y., Hassan almalki W., Rawat S., Afzal O., Alfawaz Altamimi A.S., Kazmi I., Al-Abbasi F.A., Alzarea S.I., Singh S.K. (2022). Gut microbiota disruption in COVID-19 or post-COVID illness association with severity biomarkers: A possible role of pre/pro-biotics in manipulating microflora. Chem. Biol. Interact..

[27] Carding S., Verbeke K., Vipond D.T., Corfe B.M., Owen L.J. (2015). Dysbiosis of the gut microbiota in disease. Microb. Ecol. Health Dis..

[28] Yeoh Y.K., Zuo T., Lui G.C.-Y., Zhang F., Liu Q., Li A.Y.L., Chung A.C.K., Cheung C.P., Tso E.Y.K., Fung K.S.C. (2021). Gut microbiota composition reflects disease severity and dysfunctional immune responses in patients with COVID-19. Gut.

[29] Siah K.T.H., Mahadeva S. (2022). Post-COVID-19 functional gastrointestinal disorders: Prepare for a GI aftershock. J. Gastroenterol. Hepatol..

[30] Bansal R., Gubbi S., Koch C.A. (2022). COVID-19 and chronic fatigue syndrome: An endocrine perspective. J. Clin. Transl. Endocr..

[31] Ceban F., Ling S., Lui L.M.W., Lee Y., Gill H., Teopiz K.M., Rodrigues N.B., Subramaniapillai M., Di Vincenzo J.D., Cao B. (2022). Fatigue and cognitive impairment in Post-COVID-19 Syndrome: A systematic review and meta-analysis. Brain Behav. Immun..

[32] François I.E.J.A., Lescroart O., Veraverbeke W.S., Windey K., Verbeke K., Broekaert W.F. (2014). Tolerance and the effect of high doses of wheat bran extract, containing arabinoxylan–oligosaccharides, and oligofructose on faecal output: A double-blind, randomised, placebo-controlled, cross-over trial. J. Nutr. Sci..

[33] Pansai N., Detarun P., Chinnaworn A., Sangsupawanich P., Wichienchot S. (2023). Effects of dragon fruit oligosaccharides on immunity, gut microbiome, and their metabolites in healthy adults—A randomized double-blind placebo controlled study. Food Res. Int..

[34] Faber W.X.M., Nachtegaal J., Stolwijk-Swuste J.M., Achterberg-Warmer W.J., Koning C.J.M., Besseling-van der Vaart I., van Bennekom C.A.M. (2020). Study protocol of a double-blind randomised placebo-controlled trial on the effect of a multispecies probiotic on the incidence of antibiotic-associated diarrhoea in persons with spinal cord injury. Spinal Cord.

[35] Velayati A., Kareem I., Sedaghat M., Sohrab G., Nikpayam O., Hedayati M., Abhari K., Hejazi E. (2021). Does symbiotic supplementation which contains *Bacillus Coagulans Lactobacillus rhamnosus*, *Lactobacillus acidophilus* and fructooligosaccharide has favourite effects in patients with type-2 diabetes? A randomised, double-blind, placebo-controlled trial. Arch. Physiol. Biochem..

[36] Buigues C., Fernández-Garrido J., Pruimboom L., Hoogland A.J., Navarro-Martínez R., Martínez-Martínez M., Verdejo Y., Mascarós M.C., Peris C., Cauli O. (2016). Effect of a prebiotic formulation on frailty syndrome: A randomized, double-blind clinical trial. Int. J. Mol. Sci..

[37] Neyrinck A.M., Rodriguez J., Taminiau B., Amadieu C., Herpin F., Allaert F.-A., Cani P.D., Daube G., Bindels L.B., Delzenne N.M. (2021). Improvement of gastrointestinal discomfort and inflammatory status by a synbiotic in middle-aged adults: A double-blind randomized placebo-controlled trial. Sci. Rep..

[38] Vulevic J., Tzortzis G., Juric A., Gibson G.R. (2018). Effect of a prebiotic galactooligosaccharide mixture (B-GOS^®^) on gastrointestinal symptoms in adults selected from a general population who suffer with bloating, abdominal pain, or flatulence. Neurogastroenterol. Motil..

[39] Mego M., Manichanh C., Accarino A., Campos D., Pozuelo M., Varela E., Vulevic J., Tzortzis G., Gibson G., Guarner F. (2017). Metabolic adaptation of colonic microbiota to galactooligosaccharides: A proof-of-concept-study. Aliment. Pharmacol. Ther..

[40] Depeint F., Tzortzis G., Vulevic J., I’Anson K., Gibson G.R. (2008). Prebiotic evaluation of a novel galactooligosaccharide mixture produced by the enzymatic activity of *Bifidobacterium bifidum* NCIMB 41171, in healthy humans: A randomized, double-blind, crossover, placebo-controlled intervention study. Am. J. Clin. Nutr..

[41] Childs C.E., Röytiö H., Alhoniemi E., Fekete A.A., Forssten S.D., Hudjec N., Lim Y.N., Steger C.J., Yaqoob P., Tuohy K.M. (2014). Xylo-oligosaccharides alone or in synbiotic combination with *Bifidobacterium animalis* subsp. lactis induce bifidogenesis and modulate markers of immune function in healthy adults: A double-blind, placebo-controlled, randomised, factorial cross-over study. Br. J. Nutr..

[42] Monira S., Nakamura S., Gotoh K., Izutsu K., Watanabe H., Alam N.H., Nakaya T., Horii T., Ali S.I., Iida T. (2013). Metagenomic profile of gut microbiota in children during cholera and recovery. Gut Pathog..

[43] Kau A.L., Ahern P.P., Griffin N.W., Goodman A.L., Gordon J.I. (2011). Human nutrition, the gut microbiome and the immune system. Nature.

[44] Luo J., Liang S., Jin F. (2021). Gut microbiota in antiviral strategy from bats to humans: A missing link in COVID-19. Sci. China Life Sci..

[45] Zhang X., Chen B.-D., Zhao L.-D., Li H. (2020). The gut microbiota: Emerging evidence in autoimmune diseases. Trends Mol. Med..

[46] Cantorna M.T., Snyder L., Arora J. (2019). Vitamin A and vitamin D regulate the microbial complexity, barrier function, and the mucosal immune responses to ensure intestinal homeostasis. Crit. Rev. Biochem. Mol. Biol..

[47] Trøseid M., Holter J.C., Holm K., Vestad B., Sazonova T., Granerud B.K., Dyrhol-Riise A.M., Holten A.R., Tonby K., Kildal A.B. (2023). Gut microbiota composition during hospitalization is associated with 60-day mortality after severe COVID-19. Crit. Care.

[48] Liu Q., Mak J.W.Y., Su Q., Yeoh Y.K., Lui G.C.-Y., Ng S.S.S., Zhang F., Li A.Y.L., Lu W., Hui D.S.-C. (2022). Gut microbiota dynamics in a prospective cohort of patients with post-acute COVID-19 syndrome. Gut.

[49] Xie X.-T., Zheng L.-X., Duan H.-M., Liu Y., Chen X.-Q., Cheong K.-L. (2021). Structural characteristics of *Gracilaria lemaneiformis* oligosaccharides and their alleviation of dextran sulphate sodium-induced colitis by modulating the gut microbiota and intestinal metabolites in mice. Food Funct..

[50] Zhang X., Aweya J.J., Huang Z.-X., Kang Z.-Y., Bai Z.-H., Li K.-H., He X.-T., Liu Y., Chen X.-Q., Cheong K.-L. (2020). *In vitro* fermentation of *Gracilaria lemaneiformis* sulfated polysaccharides and its agaro-oligosaccharides by human fecal inocula and its impact on microbiota. Carbohydr. Polym..

[51] Zhang X., Liu Y., Chen X.-Q., Aweya J.J., Cheong K.-L. (2020). Catabolism of *Saccharina japonica* polysaccharides and oligosaccharides by human fecal microbiota. LWT.

[52] Wang Y., Li N., Yang J.-J., Zhao D.-M., Chen B., Zhang G.-Q., Chen S., Cao R.-F., Yu H., Zhao C.-Y. (2020). Probiotics and fructo-oligosaccharide intervention modulate the microbiota-gut brain axis to improve autism spectrum reducing also the hyper-serotonergic state and the dopamine metabolism disorder. Pharmacol. Res..

[53] Zuo T., Zhang F., Lui G.C.Y., Yeoh Y.K., Li A.Y.L., Zhan H., Wan Y., Chung A.C.K., Cheung C.P., Chen N. (2020). Alterations in gut microbiota of patients with COVID-19 during time of hospitalization. Gastroenterology.

[54] Wardman J.F., Bains R.K., Rahfeld P., Withers S.G. (2022). Carbohydrate-active enzymes (CAZymes) in the gut microbiome. Nat. Rev. Microbiol..

[55] Hao Z., Wang X., Yang H., Tu T., Zhang J., Luo H., Huang H., Su X. (2021). PUL-mediated plant cell wall polysaccharide utilization in the gut *Bacteroidetes*. Int. J. Mol. Sci..

[56] Zafar H., Saier M.H. (2021). Gut *Bacteroides* species in health and disease. Gut Microbes.

[57] Mills R.H., Dulai P.S., Vázquez-Baeza Y., Sauceda C., Daniel N., Gerner R.R., Batachari L.E., Malfavon M., Zhu Q., Weldon K. (2022). Multi-omics analyses of the ulcerative colitis gut microbiome link *Bacteroides vulgatus* proteases with disease severity. Nat. Microbiol..

[58] Yang J.Y., Lee Y.S., Kim Y., Lee S.H., Ryu S., Fukuda S., Hase K., Yang C.S., Lim H.S., Kim M.S. (2017). Gut commensal *Bacteroides acidifaciens* prevents obesity and improves insulin sensitivity in mice. Mucosal Immunol..

[59] Boll E.V.J., Ekström L.M.N.K., Courtin C.M., Delcour J.A., Nilsson A.C., Björck I.M.E., Östman E.M. (2016). Effects of wheat bran extract rich in arabinoxylan oligosaccharides and resistant starch on overnight glucose tolerance and markers of gut fermentation in healthy young adults. Eur. J. Nutr..

[60] Cheong K.-L., Yu B., Chen J., Zhong S. (2022). A comprehensive review of the cardioprotective effect of marine algae polysaccharide on the gut microbiota. Foods.

[61] Desai M.S., Seekatz A.M., Koropatkin N.M., Kamada N., Hickey C.A., Wolter M., Pudlo N.A., Kitamoto S., Terrapon N., Muller A. (2016). A dietary fiber-deprived gut microbiota degrades the colonic mucus barrier and enhances pathogen susceptibility. Cell.

[62] Schwalm N.D., Groisman E.A. (2017). Navigating the gut buffet: Control of polysaccharide utilization in *Bacteroides* spp.. Trends Microbiol..

[63] Vulevic J., Juric A., Walton G.E., Claus S.P., Tzortzis G., Toward R.E., Gibson G.R. (2015). Influence of galacto-oligosaccharide mixture (B-GOS) on gut microbiota, immune parameters and metabonomics in elderly persons. Br. J. Nutr..

[64] Bindels L.B., Neyrinck A.M., Salazar N., Taminiau B., Druart C., Muccioli G.G., François E., Blecker C., Richel A., Daube G. (2015). Non digestible oligosaccharides modulate the gut microbiota to control the development of leukemia and associated cachexia in mice. PLoS ONE.

[65] Sikalidis A.K., Maykish A. (2020). The gut microbiome and type 2 diabetes mellitus: Discussing a complex relationship. Biomedicines.

[66] Gomes A.C., Hoffmann C., Mota J.F. (2018). The human gut microbiota: Metabolism and perspective in obesity. Gut Microbes.

[67] Reinold J., Farahpour F., Fehring C., Dolff S., Konik M., Korth J., van Baal L., Hoffmann D., Buer J., Witzke O. (2021). A pro-inflammatory gut microbiome characterizes SARS-CoV-2 infected patients and a reduction in the connectivity of an anti-Inflammatory bacterial network associates with severe COVID-19. Front. Cell Infect. Mi..

[68] Lindstad L.J., Lo G., Leivers S., Lu Z., Michalak L., Pereira G.V., Røhr Å.K., Martens E.C., McKee L.S., Louis P. (2021). Human gut Faecalibacterium prausnitzii deploys a highly efficient conserved system to cross-feed on β-mannan-derived oligosaccharides. mBio.

[69] Turroni F., Milani C., Duranti S., Mahony J., van Sinderen D., Ventura M. (2018). Glycan Utilization and Cross-Feeding Activities by Bifidobacteria. Trends Microbiol..

[70] Leth M.L., Ejby M., Workman C., Ewald D.A., Pedersen S.S., Sternberg C., Bahl M.I., Licht T.R., Aachmann F.L., Westereng B. (2018). Differential bacterial capture and transport preferences facilitate co-growth on dietary xylan in the human gut. Nat. Microbiol..

[71] Mary P.R., Kapoor M. (2022). Co-culture fermentations suggest cross-feeding among *Bacteroides ovatus* DSMZ 1896, *Lactiplantibacillus plantarum* WCFS1 and *Bifidobacterium adolescentis* DSMZ 20083 for utilizing dietary galactomannans. Food Res. Int..

[72] Wang M., Cheong K.-L. (2023). Preparation, structural characterisation, and bioactivities of fructans: A review. Molecules.

[73] Yao W., Gong Y., Li L., Hu X., You L. (2022). The effects of dietary fibers from rice bran and wheat bran on gut microbiota: An overview. Food Chem. X.

[74] Hu J., Lin S., Zheng B., Cheung P.C.K. (2018). Short-chain fatty acids in control of energy metabolism. Crit. Rev. Food Sci. Nutr..

[75] Martin-Gallausiaux C., Marinelli L., Blottière H.M., Larraufie P., Lapaque N. (2020). SCFA: Mechanisms and functional importance in the gut. Proc. Nutr. Soc..

[76] Dalile B., Van Oudenhove L., Vervliet B., Verbeke K. (2019). The role of short-chain fatty acids in microbiota–gut–brain communication. Nat. Rev. Gastro. Hepat..

[77] Yao Y., Cai X., Fei W., Ye Y., Zhao M., Zheng C. (2022). The role of short-chain fatty acids in immunity, inflammation and metabolism. Crit. Rev. Food Sci. Nutr..

[78] Zhang F., Wan Y., Zuo T., Yeoh Y.K., Liu Q., Zhang L., Zhan H., Lu W., Xu W., Lui G.C.Y. (2022). Prolonged impairment of short-chain fatty acid and L-isoleucine biosynthesis in gut microbiome in patients with COVID-19. Gastroenterology.

[79] Giovanni M., Cesare C., Maria Raffaella B., Giulia C., Francesca F., Anna K., Dmitry B., Vasile D., Egidia M., Pietro F. (2023). Post COVID-19 irritable bowel syndrome. Gut.

[80] Zhang F., Lau R.I., Liu Q., Su Q., Chan F.K.L., Ng S.C. (2022). Gut microbiota in COVID-19: Key microbial changes, potential mechanisms and clinical applications. Nat. Rev. Gastro. Hepat..

[81] Hotchkiss A.T., Renye J.A., White A.K., Nunez A., Guron G.K.P., Chau H., Simon S., Poveda C., Walton G., Rastall R. (2022). Cranberry arabino-xyloglucan and pectic oligosaccharides induce *Lactobacillus* growth and short-chain fatty acid production. Microorganisms.

[82] Godínez-Méndez L.A., Gurrola-Díaz C.M., Zepeda-Nuño J.S., Vega-Magaña N., Lopez-Roa R.I., Íñiguez-Gutiérrez L., García-López P.M., Fafutis-Morris M., Delgado-Rizo V. (2021). In iivo healthy benefits of galacto-oligosaccharides from *Lupinus albus* (LA-GOS) in butyrate production through intestinal microbiota. Biomolecules.

[83] Haase S., Haghikia A., Wilck N., Müller D.N., Linker R.A. (2018). Impacts of microbiome metabolites on immune regulation and autoimmunity. Immunology.

[84] Abdalkareem Jasim S., Jade Catalan Opulencia M., Alexis Ramírez-Coronel A., Kamal Abdelbasset W., Hasan Abed M., Markov A., Raheem Lateef Al-Awsi G., Azamatovich Shamsiev J., Thaeer Hammid A., Nader Shalaby M. (2022). The emerging role of microbiota-derived short-chain fatty acids in immunometabolism. Int. Immunopharmacol..

[85] Qiu J., Villa M., Sanin D.E., Buck M.D., O’Sullivan D., Ching R., Matsushita M., Grzes K.M., Winkler F., Chang C.-H. (2019). Acetate promotes T cell effector function during glucose restriction. Cell Rep..

[86] Yong S.J. (2021). Long COVID or post-COVID-19 syndrome: Putative pathophysiology, risk factors, and treatments. Infect. Dis..

[87] Andrade M.E.R., Trindade L.M., Leocádio P.C.L., Leite J.I.A., dos Reis D.C., Cassali G.D., da Silva T.F., de Oliveira Carvalho R.D., de Carvalho Azevedo V.A., Cavalcante G.G. (2023). Association of fructo-oligosaccharides and arginine improves severity of mucositis and modulate the intestinal microbiota. Probiotics Antimicrob. Proteins.

[88] Trompette A., Gollwitzer E.S., Pattaroni C., Lopez-Mejia I.C., Riva E., Pernot J., Ubags N., Fajas L., Nicod L.P., Marsland B.J. (2018). Dietary fiber confers protection against flu by shaping Ly6c− patrolling monocyte hematopoiesis and CD8+ T cell metabolism. Immunity.

[89] Xu J., Wang R., Liu W., Yin Z., Wu J., Yu X., Wang W., Zhang H., Li Z., Gao M. (2023). The specificity of ten non-digestible carbohydrates to enhance butyrate-producing bacteria and butyrate production in vitro fermentation. Food Sci. Hum. Well..

[90] Guo C., Che X., Briese T., Ranjan A., Allicock O., Yates R.A., Cheng A., March D., Hornig M., Komaroff A.L. (2023). Deficient butyrate-producing capacity in the gut microbiome is associated with bacterial network disturbances and fatigue symptoms in ME/CFS. Cell Host Microbe.

[91] Zhang D., Zhou Y., Ma Y., Chen P., Tang J., Yang B., Li H., Liang M., Xue Y., Liu Y. (2023). Gut microbiota dysbiosis correlates with long COVID-19 at one-year after discharge. J. Korean Med. Sci..

[92] Xie Q., Sun Y., Cao L., Chen L., Chen J., Cheng X., Wang C. (2020). Antifatigue and antihypoxia activities of oligosaccharides and polysaccharides from *Codonopsis pilosula* in mice. Food Funct..

[93] Zhuang Y., Zeng R., Liu X., Yang L., Chan Z. (2022). Neoagaro-oligosaccharides ameliorate chronic restraint stress-induced depression by increasing 5-HT and BDNF in the brain and remodeling the gut microbiota of mice. Mar. Drugs.

[94] Barrea L., Grant W.B., Frias-Toral E., Vetrani C., Verde L., de Alteriis G., Docimo A., Savastano S., Colao A., Muscogiuri G. (2022). Dietary recommendations for post-COVID-19 syndrome. Nutrients.

[95] Hu Y.-C., Hu J.-L., Li J., Wang J., Zhang X.-Y., Wu X.-Y., Li X., Guo Z.-B., Zou L., Wu D.-T. (2023). Physicochemical characteristics and biological activities of soluble dietary fibers isolated from the leaves of different quinoa cultivars. Food Res. Int..

[96] Barrea L., Vetrani C., Caprio M., Cataldi M., Ghoch M.E., Elce A., Camajani E., Verde L., Savastano S., Colao A. (2022). From the ketogenic diet to the Mediterranean diet: The potential dietary therapy in patients with obesity after COVID-19 infection (post COVID syndrome). Curr. Obes. Rep..

[97] Pandey M., Bhati A., Priya K., Sharma K.K., Singhal B. (2022). Precision postbiotics and mental health: The management of post-COVID-19 complications. Probiotics Antimicrob. Proteins.

[98] Buffie C.G., Bucci V., Stein R.R., McKenney P.T., Ling L., Gobourne A., No D., Liu H., Kinnebrew M., Viale A. (2015). Precision microbiome reconstitution restores bile acid mediated resistance to *Clostridium difficile*. Nature.

[99] Henri D., Sylvie R., Dominique R., David B., Marie-Anne M., Elodie Q., Ginette T., Véronique B., Lydie H., Guillaume D. (2013). Connecting dysbiosis, bile-acid dysmetabolism and gut inflammation in inflammatory bowel diseases. Gut.

[100] Ward J.B.J., Lajczak N.K., Kelly O.B., O’Dwyer A.M., Giddam A.K., Gabhann J.N., Franco P., Tambuwala M.M., Jefferies C.A., Keely S. (2017). Ursodeoxycholic acid and lithocholic acid exert anti-inflammatory actions in the colon. Am. J. Physiol.-Gastrointest. Liver Physiol..

[101] Singh N., Gurav A., Sivaprakasam S., Brady E., Padia R., Shi H., Thangaraju M., Prasad P.D., Manicassamy S., Munn D.H. (2014). Activation of Gpr109a, receptor for niacin and the commensal metabolite butyrate, suppresses colonic inflammation and carcinogenesis. Immunity.

[102] Braniste V., Al-Asmakh M., Kowal C., Anuar F., Abbaspour A., Tóth M., Korecka A., Bakocevic N., Ng L.G., Kundu P. (2014). The gut microbiota influences blood-brain barrier permeability in mice. Sci. Transl. Med..

[103] Erny D., Hrabě de Angelis A.L., Jaitin D., Wieghofer P., Staszewski O., David E., Keren-Shaul H., Mahlakoiv T., Jakobshagen K., Buch T. (2015). Host microbiota constantly control maturation and function of microglia in the CNS. Nat. Neurosci..

[104] Thorburn A.N., McKenzie C.I., Shen S., Stanley D., Macia L., Mason L.J., Roberts L.K., Wong C.H.Y., Shim R., Robert R. (2015). Evidence that asthma is a developmental origin disease influenced by maternal diet and bacterial metabolites. Nat. Commun..

[105] Trompette A., Gollwitzer E.S., Yadava K., Sichelstiel A.K., Sprenger N., Ngom-Bru C., Blanchard C., Junt T., Nicod L.P., Harris N.L. (2014). Gut microbiota metabolism of dietary fiber influences allergic airway disease and hematopoiesis. Nat. Med..

[106] Connors J., Dunn K.A., Allott J., Bandsma R., Rashid M., Otley A.R., Bielawski J.P., Van Limbergen J. (2020). The relationship between fecal bile acids and microbiome community structure in pediatric Crohn’s disease. ISME J..

[107] Winston J.A., Theriot C.M. (2020). Diversification of host bile acids by members of the gut microbiota. Gut Microbes.

[108] Kong F., Saif L.J., Wang Q. (2021). Roles of bile acids in enteric virus replication. Anim. Dis..

[109] Castañé H., Iftimie S., Baiges-Gaya G., Rodríguez-Tomàs E., Jiménez-Franco A., López-Azcona A.F., Garrido P., Castro A., Camps J., Joven J. (2022). Machine learning and semi-targeted lipidomics identify distinct serum lipid signatures in hospitalized COVID-19-positive and COVID-19-negative patients. Metabolism.

[110] Hoving L.R., Katiraei S., Heijink M., Pronk A., van der Wee-Pals L., Streefland T., Giera M., Willems van Dijk K., van Harmelen V. (2018). Dietary mannan oligosaccharides modulate gut microbiota, increase fecal bile acid excretion, and decrease plasma cholesterol and atherosclerosis development. Mol. Nutr. Food Res..

[111] Li Y., Tian Y., Cai W., Wang Q., Chang Y., Sun Y., Dong P., Wang J. (2021). Novel ι-carrageenan tetrasaccharide alleviates liver lipid accumulation via the bile acid–FXR–SHP/PXR pathway to regulate cholesterol conversion and fatty acid metabolism in insulin-resistant mice. J. Agric. Food. Chem..

[112] Sinha S.R., Haileselassie Y., Nguyen L.P., Tropini C., Wang M., Becker L.S., Sim D., Jarr K., Spear E.T., Singh G. (2020). Dysbiosis-Induced Secondary Bile Acid Deficiency Promotes Intestinal Inflammation. Cell Host Microbe.

[113] Ko W.-K., Lee S.-H., Kim S.J., Jo M.-J., Kumar H., Han I.-B., Sohn S. (2017). Anti-inflammatory effects of ursodeoxycholic acid by lipopolysaccharide-stimulated inflammatory responses in RAW 264.7 macrophages. PLoS ONE.

[114] Calmus Y., Weill B., Ozier Y., Chéreau C., Houssin D., Poupon R. (1992). Immunosuppressive properties of chenodeoxycholic and ursodeoxycholic acids in the mouse. Gastroenterology.

[115] Abdulrab S., Al-Maweri S., Halboub E. (2020). Ursodeoxycholic acid as a candidate therapeutic to alleviate and/or prevent COVID-19-associated cytokine storm. Med. Hypotheses.

[116] Wahlström A., Sayin S.I., Marschall H.-U., Bäckhed F. (2016). Intestinal crosstalk between bile acids and microbiota and its impact on host metabolism. Cell Metab..

[117] Ding L., Yang L., Wang Z., Huang W. (2015). Bile acid nuclear receptor FXR and digestive system diseases. Acta Pharm. Sin. B.

[118] Brevini T., Maes M., Webb G.J., John B.V., Fuchs C.D., Buescher G., Wang L., Griffiths C., Brown M.L., Scott W.E. (2023). FXR inhibition may protect from SARS-CoV-2 infection by reducing ACE2. Nature.

[119] Hollman D.A.A., Milona A., van Erpecum K.J., van Mil S.W.C. (2012). Anti-inflammatory and metabolic actions of FXR: Insights into molecular mechanisms. Biochim. Et Biophys. Acta (BBA)-Mol. Cell Biol. Lipids.

[120] Batiha G.E.-S., Al-kuraishy H.M., Al-Gareeb A.I., Youssef F.S., El-Sherbeni S.A., Negm W.A. (2023). A perspective study of the possible impact of obeticholic acid against SARS-CoV-2 infection. Inflammopharmacology.

[121] Li M.-Y., Li L., Zhang Y., Wang X.-S. (2020). Expression of the SARS-CoV-2 cell receptor gene ACE2 in a wide variety of human tissues. Infect. Dis. Poverty.

[122] Chaisuwan W., Phimolsiripol Y., Chaiyaso T., Techapun C., Leksawasdi N., Jantanasakulwong K., Rachtanapun P., Wangtueai S., Sommano S.R., You S. (2021). The antiviral activity of bacterial, fungal, and algal polysaccharides as bioactive ingredients: Potential uses for enhancing immune systems and preventing viruses. Front. Nutr..

[123] Gupta Y., Maciorowski D., Zak S.E., Kulkarni C.V., Herbert A.S., Durvasula R., Fareed J., Dye J.M., Kempaiah P. (2021). Heparin: A simplistic repurposing to prevent SARS-CoV-2 transmission in light of its *in-vitro* nanomolar efficacy. Int. J. Biol. Macromol..

[124] Kwon P.S., Oh H., Kwon S.-J., Jin W., Zhang F., Fraser K., Hong J.J., Linhardt R.J., Dordick J.S. (2020). Sulfated polysaccharides effectively inhibit SARS-CoV-2 in vitro. Cell Discov..

[125] Chittum J.E., Sankaranarayanan N.V., O’Hara C.P., Desai U.R. (2021). On the selectivity of heparan sulfate recognition by SARS-CoV-2 spike glycoprotein. ACS Med. Chem. Lett..

[126] Hao W., Ma B., Li Z., Wang X., Gao X., Li Y., Qin B., Shang S., Cui S., Tan Z. (2021). Binding of the SARS-CoV-2 spike protein to glycans. Sci. Bull..

[127] Villapol S. (2020). Gastrointestinal symptoms associated with COVID-19: Impact on the gut microbiome. Transl. Res..

[128] Yang T., Chakraborty S., Saha P., Mell B., Cheng X., Yeo J.-Y., Mei X., Zhou G., Mandal J., Golonka R. (2020). Gnotobiotic rats reveal that gut microbiota regulates colonic mRNA of Ace2, the receptor for SARS-CoV-2 infectivity. Hypertension.

[129] He F., Zhang T., Xue K., Fang Z., Jiang G., Huang S., Li K., Gu Z., Shi H., Zhang Z. (2021). Fecal multi-omics analysis reveals diverse molecular alterations of gut ecosystem in COVID-19 patients. Anal. Chim. Acta.

[130] Saint-Criq V., Lugo-Villarino G., Thomas M. (2021). Dysbiosis, malnutrition and enhanced gut-lung axis contribute to age-related respiratory diseases. Ageing Res. Rev..

[131] Budden K.F., Gellatly S.L., Wood D.L.A., Cooper M.A., Morrison M., Hugenholtz P., Hansbro P.M. (2017). Emerging pathogenic links between microbiota and the gut–lung axis. Nat. Rev. Microbiol..

[132] Olímpio F., Andreata-Santos R., Rosa P.C., Santos W., Oliveira C., Aimbire F. (2022). *Lactobacillus rhamnosus* restores antiviral signaling and attenuates cytokines secretion from human bronchial epithelial cells exposed to cigarette smoke and infected with SARS-CoV-2. Probiotics Antimicrob. Proteins.

[133] Manna S., Chowdhury T., Chakraborty R., Mandal S.M. (2021). Probiotics-derived peptides and their immunomodulatory molecules can play a preventive role against viral diseases including COVID-19. Probiotics Antimicrob. Proteins.

[134] Brown J.A., Sanidad K.Z., Lucotti S., Lieber C.M., Cox R.M., Ananthanarayanan A., Basu S., Chen J., Shan M., Amir M. (2022). Gut microbiota-derived metabolites confer protection against SARS-CoV-2 infection. Gut Microbes.

[135] Li J., Richards E.M., Handberg E.M., Pepine C.J., Raizada M.K. (2021). Butyrate regulates COVID-19–relevant genes in gut epithelial organoids from normotensive rats. Hypertension.

